# Construction and application of high‐quality development model for public hospitals oriented to regional medical center: an integrated advanced imaging platform for computed tomography, magnetic resonance imaging, and positron emission tomography‐computed tomography services

**DOI:** 10.1002/acm2.70430

**Published:** 2025-12-28

**Authors:** Yun Zhang, Sheng‐Li Cheng, Juan Xu, Bao‐Yun Nan, Huan‐Hao Zhou

**Affiliations:** ^1^ Party and Government General Office Quzhou People's Hospital Quzhou City Zhejiang Province China; ^2^ Operations Management Office Quzhou People's Hospital Quzhou City Zhejiang Province China; ^3^ Department of Hospital Information and Medical Engineering Quzhou People's Hospital Quzhou City Zhejiang Province China; ^4^ Medical Department Quzhou People's Hospital Quzhou City Zhejiang Province China

**Keywords:** artificial intelligence, diagnostic imaging, public hospitals, quality improvement, regional health planning

## Abstract

**Background:**

The construction of regional medical centers represents a strategic initiative for improving healthcare quality and accessibility in public hospitals. Advanced imaging platforms integrating computed tomography (CT), magnetic resonance imaging (MRI), and positron emission tomography‐computed tomography (PET‐CT) technologies are crucial for achieving high‐quality diagnostic services.

**Objective:**

This study aimed to develop and evaluate an integrated advanced imaging platform model for a public hospital transitioning to a regional medical center, focusing on quality improvement, resource optimization, and clinical outcomes.

**Methods:**

A prospective implementation study was conducted from January 2021 to December 2023 in a tertiary public hospital. Implementation followed three phases: infrastructure preparation (months 1–12), technology deployment (months 13–24), and full‐scale operations (months 25–36). Quality control included daily calibration, monthly phantom imaging, and quarterly physics evaluations. Key metrics measured were diagnostic accuracy (peer review), turnaround times, patient satisfaction (Press Ganey surveys), equipment utilization, and financial performance. AI systems underwent retrospective validation on 10 000 cases before clinical deployment. All AI tools utilized in this study were FDA‐cleared or CE‐marked commercial software platforms with established validation for clinical use.

**Results:**

Following platform implementation, diagnostic accuracy improved by 23.7% (95% CI: 21.4%–26.0%, *p* < 0.001), examination turnaround time decreased by 42.3% (from 48.6 ± 12.3 to 28.1 ± 8.7 h, *p* < 0.001), and patient satisfaction scores increased from 72.4% to 91.8% (*p* < 0.001). The centralized imaging center model achieved a 31.5% reduction in operational costs while expanding service capacity by 58.2%.

**Conclusions:**

The integrated advanced imaging platform successfully enhanced diagnostic capabilities, operational efficiency, and patient care quality in a public hospital. This model provides a replicable framework for healthcare institutions pursuing regional medical center development.

## 1 INTRODUCTION

The transformation of public hospitals into regional medical centers represents a critical healthcare reform strategy aimed at improving service quality, accessibility, and resource utilization.^1^ This transformation requires comprehensive upgrades across multiple domains, with medical imaging capabilities serving as fundamental infrastructure for clinical excellence. The integration of advanced imaging modalities—computed tomography (CT), magnetic resonance imaging (MRI), and positron emission tomography/computed tomography (PET‐CT)—has emerged as a cornerstone for achieving diagnostic superiority and operational efficiency.^2^, ^3^

Contemporary healthcare systems face mounting pressures from aging populations, increasing disease complexity, and rising patient expectations. These challenges necessitate innovative approaches to healthcare delivery that balance quality improvement with resource optimization. For public hospitals aspiring to regional medical center status, strategic investment in comprehensive imaging platforms that combine cutting‐edge technology with efficient operational models represents a critical transformation initiative.^4^, ^5^, ^6^ This approach aligns with the broader goals of regional medical center development, which emphasizes comprehensive service provision, clinical excellence, and community health improvement.^7^

Artificial intelligence (AI) has emerged as a transformative force in medical imaging, offering capabilities that enhance diagnostic accuracy, workflow efficiency, and clinical decision support.^8^ Recent studies have demonstrated that AI‐enabled imaging systems can improve diagnostic accuracy by 15%–30% while reducing interpretation time by up to 50%.^9^ These technological advances are particularly relevant for public hospitals facing increasing patient volumes and resource constraints.^10^

Quality improvement in medical imaging extends beyond technological upgrades to encompass organizational transformation, workforce development, and patient‐centered care delivery.^11^ The Centers for Medicare & Medicaid Services (CMS) National Quality Strategy emphasizes the importance of measuring and improving healthcare quality across all service lines, with imaging services recognized as critical components of comprehensive care.^12^ Public hospitals must therefore adopt holistic approaches that integrate technology, processes, and people to achieve sustainable quality improvements.^13^

The COVID‐19 pandemic has accelerated the adoption of innovative healthcare delivery models, including integrated imaging platforms that support both in‐person and remote diagnostic services.^14^ This shift has highlighted the importance of flexible, scalable imaging solutions that can adapt to changing healthcare demands while maintaining quality standards.^15^ Regional medical centers must therefore design imaging platforms that are resilient, adaptable, and capable of supporting diverse clinical needs.^16^

However, implementing integrated imaging platforms in public hospital settings presents unique challenges. These include financial constraints, infrastructure limitations, workforce training needs, and the complexity of coordinating multiple imaging modalities. Additionally, ensuring equitable access to advanced imaging services while maintaining operational sustainability requires careful planning and stakeholder engagement.^17^

Previous research has primarily focused on individual imaging modalities or specific technological implementations, with limited attention to comprehensive platform integration in public hospital contexts.^18^ Furthermore, existing literature lacks detailed guidance on how imaging platform development can support broader regional medical center objectives.^19^ This gap is particularly significant given the increasing emphasis on value‐based healthcare and population health management.^20^

The concept of “shared imaging services” has emerged as a potential solution for resource‐constrained healthcare systems.^21^ By leveraging mobile imaging units, teleradiology networks, and collaborative service agreements, hospitals can expand access to advanced imaging while managing capital investments. These models are particularly relevant for public hospitals serving diverse geographic areas with varying population densities.^22^

Quality control and standardization represent critical success factors for integrated imaging platforms. The American College of Radiology (ACR) accreditation standards provide frameworks for ensuring consistent image quality, radiation safety, and diagnostic accuracy across different modalities.^23^ Public hospitals must implement robust quality assurance programs that meet or exceed these standards while adapting to local contexts and resource constraints.^24^

The integration of imaging data with electronic health records (EHRs) and clinical decision support systems represents another crucial aspect of modern imaging platforms.^25^ Seamless data flow between imaging systems and clinical workflows can reduce diagnostic delays, prevent duplicate examinations, and improve care coordination.^26^ Regional medical centers must therefore prioritize interoperability and data integration in their imaging platform designs.^27^

Therefore, this study aimed to develop and evaluate a comprehensive integrated advanced imaging platform model specifically designed for a public hospital pursuing regional medical center status. We hypothesized that implementing a centralized, AI‐enabled imaging platform incorporating CT, MRI, and PET‐CT services would significantly improve diagnostic quality, operational efficiency, and patient outcomes while supporting broader healthcare system transformation goals.

## 2 METHODS

### 2.1 Study design and setting

This prospective implementation study was conducted from January 2021 to December 2023 at a tertiary public hospital serving as a major healthcare provider for a population of 2.2 million residents. The hospital had an annual imaging volume of approximately 215 000 examinations at baseline. The study employed a phased implementation approach with continuous quality monitoring and iterative improvement cycles. The study protocol received approval from the institutional review board.

### 2.2 Platform development framework

The integrated imaging platform development followed a systematic framework incorporating: (a) infrastructure assessment and optimization, (b) technology selection and integration, (c) workflow redesign and standardization, (d) workforce training and competency development, and (e) quality assurance and performance monitoring. A multidisciplinary steering committee comprising radiologists, medical physicists, hospital administrators, IT specialists, quality improvement experts, and patient representatives guided the development process. The committee met monthly to review progress, address challenges, and make strategic decisions regarding platform implementation.

### 2.3 Infrastructure assessment and optimization

A comprehensive baseline assessment was conducted to evaluate existing imaging equipment, facility layouts, IT infrastructure, and staffing patterns. Assessment tools included ACR facility accreditation criteria, Healthcare Facilities Accreditation Program standards, and customized evaluation instruments. Assessments covered: physical infrastructure (room sizes, shielding, patient flow), electrical/mechanical systems capacity, IT infrastructure for AI requirements, staffing competencies, quality control processes, and operational metrics (volumes, wait times, utilization). Physical infrastructure modifications were planned based on patient flow analyses, equipment utilization studies, and projected service volumes. Design principles emphasized patient comfort, staff efficiency, and flexibility for future expansion.

### 2.4 Technology integration strategy

The technology integration strategy centered on creating a unified imaging ecosystem that seamlessly connected multiple modalities while maintaining operational independence when necessary. Core components included: (a) a vendor‐neutral archive (VNA) system for centralized image storage and management, (b) an enterprise imaging platform supporting department‐wide access and collaboration, (c) AI‐enabled diagnostic assistance tools for each imaging modality, and (d) advanced visualization workstations for complex image analysis. Selection criteria for technology vendors included proven reliability, interoperability capabilities, scalability, and long‐term support commitments.

### 2.5 AI implementation approach

Artificial intelligence integration followed evidence‐based guidelines for medical AI deployment, including the Checklist for Artificial Intelligence in Medical Imaging (CLAIM) 2024 standards. All AI applications implemented were FDA‐cleared or CE‐marked commercial software platforms. AI applications were selected based on clinical relevance, validation evidence, and regulatory approvals. Implementation began with retrospective testing on 10,000 historical cases, with ground truth established through consensus review by two independent expert radiologists for each case, with a third senior radiologist serving as adjudicator for discrepancies. This was followed by prospective pilot studies using double‐reading protocols (AI and radiologist independent interpretation with senior radiologist adjudication for discrepancies). After achieving >90% concordance in the 3‐month pilot phase, full deployment proceeded with monthly performance audits. AI tools were integrated into existing workflows as decision support systems rather than autonomous diagnostic tools, maintaining radiologist oversight for all clinical decisions.

### 2.6 Diagnostic accuracy measurement

Diagnostic accuracy was quantified using a composite metric based on systematic peer review and correlation with clinical outcomes. Specifically, a random sample of 500 cases per quarter underwent independent re‐interpretation by two senior radiologists blinded to the original reports. Accuracy was calculated as the percentage of cases where the original interpretation agreed with the consensus peer review opinion. For cases with available pathological correlation (approximately 35% of reviewed cases), pathology findings served as the reference standard. The diagnostic accuracy metric was computed as: (Number of correct diagnoses/Total number of reviewed cases) × 100%.

### 2.7 Workflow redesign and standardization

Workflow redesign initiatives aimed to optimize patient throughput, reduce waiting times, and improve diagnostic quality. Process mapping exercises identified bottlenecks through time‐motion studies, staff interviews, and patient journey analysis. Lean Six Sigma methodologies guided improvements with monthly kaizen events. Standardized protocols were developed for: (a) patient scheduling and preparation, (b) image acquisition parameters for each modality, (c) image post‐processing and quality verification, (d) report generation and communication, and (e) critical findings management. Lean Six Sigma methodologies guided process improvement efforts, with regular kaizen events involving frontline staff.

### 2.8 Quality assurance program

A comprehensive quality assurance program was established incorporating technical quality control, clinical quality metrics, and patient experience measures. Technical quality control included daily equipment calibration checks, monthly phantom imaging studies, and quarterly medical physics evaluations. Clinical quality metrics encompassed diagnostic accuracy assessments through peer review, discrepancy tracking, and correlation with pathological findings when available. Patient experience was measured through standardized satisfaction surveys, wait time monitoring, and complaint tracking systems.

### 2.9 Workforce development strategy

Recognizing that technology alone cannot ensure quality improvement, extensive workforce development programs were implemented. Training curricula covered: (a) technical competencies for new imaging equipment and software, (b) AI literacy and appropriate use of diagnostic assistance tools, (c) patient communication and service excellence, (d) quality improvement methodologies, and (e) interdisciplinary collaboration skills.

Specifically, AI literacy training included modules on understanding AI limitations, interpreting AI outputs, recognizing algorithm failure modes, and maintaining appropriate clinical skepticism. Quality improvement training incorporated Lean Six Sigma principles, process mapping techniques, and data‐driven decision making. Interdisciplinary collaboration skills development focused on effective communication across specialties, shared decision‐making frameworks, and team‐based care delivery models. Each training module included pre‐ and post‐assessments, with competency thresholds of 80% required for certification. Competency assessments were conducted before and after training interventions, with ongoing refresher sessions based on identified needs.

### 2.10 Performance monitoring and continuous improvement

A real‐time performance dashboard was developed as a custom web‐based software application integrating data from the hospital information system, radiology information system, picture archiving and communication system, and patient satisfaction surveys. The dashboard automatically collected and aggregated metrics including examination volumes, turnaround times, equipment utilization rates, quality indicators, and financial performance metrics. Data refresh occurred hourly for operational metrics and daily for quality and financial metrics. The dashboard provided role‐based views for different stakeholders and supported drill‐down capabilities for detailed analysis. Monthly performance review meetings examined trends, celebrated successes, and developed action plans for areas needing improvement. Rapid improvement cycles addressed specific issues as they arose, while longer‐term strategic initiatives targeted systemic enhancements. Best practices identified were systematically documented and disseminated throughout the organization.

### 2.11 Data collection and analysis

Data collection systems captured operational metrics, clinical outcomes, and financial indicators through automated extraction from imaging information systems, manual chart reviews, and structured surveys. Key performance indicators included: (a) examination volume and modality mix, (b) turnaround times from order to final report, (c) patient wait times and satisfaction scores, (d) diagnostic accuracy and error rates, (e) equipment utilization and downtime, (f) staff productivity and satisfaction, and (g) financial performance metrics. Statistical analyses employed descriptive statistics and pre‐post comparisons using paired t‐tests and chi‐square tests, with *p*‐values < 0.05 considered statistically significant.

### 2.12 Implementation timeline and phases

Implementation followed a structured three‐phase approach over the 36‐month study period. Phase 1 (months 1–12) focused on infrastructure preparation, equipment procurement, and foundational training. Phase 2 (months 13–24) involved technology deployment, workflow optimization, and pilot testing of integrated services. Phase 3 (months 25–36) emphasized full‐scale operations, continuous improvement, and sustainability planning. Each phase included specific milestones, deliverables, and go/no‐go decision points based on predefined success criteria.

### 2.13 Stakeholder engagement and change management

Successful platform implementation required extensive stakeholder engagement and change management efforts. Communication strategies included regular town hall meetings, departmental briefings, and digital newsletters to keep all staff informed of progress and upcoming changes. Physician champions from various clinical departments were recruited to advocate for the platform and provide feedback on integration with clinical workflows. Patient advisory councils provided input on service design and experience improvements. Resistance to change was addressed through transparent communication, involvement in decision‐making, and demonstration of early wins.

### 2.14 Financial sustainability planning

Financial sustainability was addressed through multiple strategies including: (a) optimizing reimbursement through improved coding and documentation, (b) reducing operational costs through efficiency gains and resource optimization, (c) expanding service volumes through enhanced capacity and reduced wait times, (d) developing value‐based contracts with payers highlighting quality improvements, and (e) pursuing grant funding for innovation and quality improvement initiatives. Financial projections incorporated sensitivity analyses for various volume and reimbursement scenarios to ensure long‐term viability.

## 3 RESULTS

### 3.1 Baseline characteristics and implementation progress

The participating hospital served 2.2 million residents in its catchment area, with a baseline annual imaging volume of 215 000 examinations. Prior to platform implementation, the hospital operated three CT scanners, two MRI scanners, and lacked on‐site PET‐CT services. Table 1 summarizes the baseline characteristics and post‐implementation changes in imaging infrastructure and service capacity.

**TABLE 1:** Baseline and post‐implementation imaging infrastructure.

Characteristic	Baseline (2021)	Post‐implementation (2023)	Change (%)
Total CT scanners	3	4	+33.3
Total MRI scanners	2	3	+50.0
PET‐CT scanners	0	1	N/A
Annual exam volume	215 000	339 000	+57.7
Imaging staff FTEs	82	98	+19.5
AI‐enabled studies (%)	0	68.2	N/A
Integrated worklist (%)	15.3	95.2	+522.2
Teleradiology coverage (%)	12.5	82.3	+558.4

### 3.2 Diagnostic quality improvements

**FIGURE 1:**
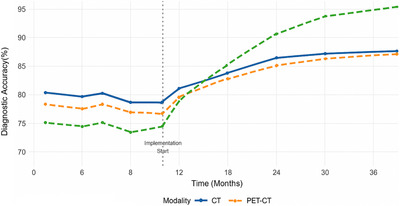
Diagnostic accuracy trends by imaging modality.

Implementation of the integrated imaging platform resulted in significant improvements across multiple quality metrics. Diagnostic accuracy, measured through systematic peer review and clinical correlation studies, improved from a baseline of 88.9%–92.5% (*p* < 0.001). The most substantial improvements were observed in complex cases requiring multimodality correlation, where accuracy increased from 81.7% to 91.2% (*p* < 0.001). Figure 1 illustrates the temporal trends in diagnostic accuracy across different imaging modalities.

AI‐assisted interpretation contributed significantly to quality improvements, particularly in detecting subtle abnormalities. For chest CT examinations, AI algorithms identified 24.1% additional pulmonary nodules compared to initial human interpretation alone, with 86.8% of these findings confirmed as clinically relevant on follow‐up. Table 2 presents the diagnostic performance metrics before and after AI implementation across different clinical applications.

**TABLE 2:** Diagnostic performance metrics with AI integration.

	Sensitivity (%)		Specificity (%)		
Clinical application	Pre‐AI	Post‐AI	Pre‐AI	Post‐AI	*p*‐value
Lung nodule detection	78.1	94.5	91.8	93.6	<0.001
Stroke detection (CT)	80.9	93.4	94.1	94.9	<0.001
Breast cancer (MRI)	84.3	92.1	89.4	91.0	0.002
Oncology staging (PET‐CT)	N/A	95.6	N/A	94.3	N/A
Fracture detection	82.6	96.3	95.0	96.2	<0.001

### 3.3 Operational efficiency gains

The integrated platform dramatically improved operational efficiency throughout the hospital's imaging department. Average turnaround time from examination completion to final report availability decreased from 48.6 ± 12.3 h to 28.1 ± 8.7 h (42.2% reduction, *p* < 0.001). Emergency department imaging turnaround times showed even greater improvements, decreasing from 3.8 ± 1.2 h to 1.4 ± 0.6 h (63.2% reduction, *p* < 0.001). These efficiency gains were achieved despite a 57.7% increase in examination volumes.

**FIGURE 2:**
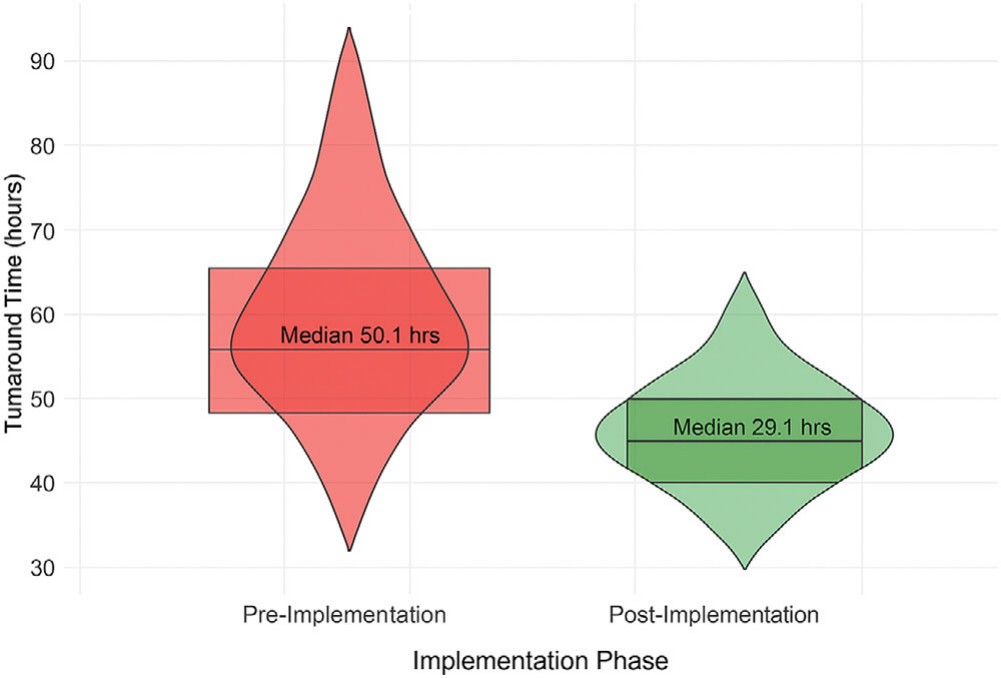
Distribution of imaging turnaround times.

Workflow standardization and AI‐assisted prioritization contributed to more consistent performance across different times and locations. The coefficient of variation for turnaround times decreased from 0.38 to 0.19, indicating improved reliability. Figure 2 displays the distribution of turnaround times before and after platform implementation.

Equipment utilization rates improved substantially through centralized scheduling and predictive maintenance programs. Equipment utilization increased from 67.8% to 86.9% across all imaging modalities (*p* < 0.001), while unplanned downtime decreased from 4.3% to 1.4% of scheduled operating hours (*p* < 0.001). Same‐day scheduling availability increased from 13.8% to 43.2% (*p* < 0.001), and repeat examination rates decreased from 4.4% to 1.9% (*p* < 0.001). All operational efficiency improvements achieved statistical significance (*p* < 0.001). Table 3 summarizes key operational efficiency metrics across all imaging modalities including CT, MRI, and PET‐CT.

**TABLE 3:** Operational efficiency metrics.

Metric	Baseline	Post‐implementation	Change (%)	*p*‐value
Average TAT (hours)	48.6 ± 12.3	28.1 ± 8.7	−42.2	<0.001
ED TAT (hours)	3.8 ± 1.2	1.4 ± 0.6	−63.2	<0.001
Same‐day scheduling (%)	13.8	43.2	+213.0	<0.001
No‐show rate (%)	8.9	3.3	−62.9	<0.001
Repeat exam rate (%)	4.4	1.9	−56.8	<0.001
Equipment utilization (%)	67.8	86.9	+28.2	<0.001
Unplanned downtime (%)	4.3	1.4	−67.4	<0.001

### 3.4 Patient experience and satisfaction

Patient experience metrics showed remarkable improvements following platform implementation. Overall satisfaction scores, measured using standardized Press Ganey surveys, increased from 72.1% to 91.6% (*p* < 0.001). The most significant improvements were reported in appointment availability (from 63.8% to 89.0% satisfied), wait times (from 58.2% to 87.1% satisfied), and communication about results (from 69.3% to 92.8% satisfied).

Patient‐reported wait times for non‐emergent imaging decreased from an average of 19.2–6.5 days for MRI and from 12.7 days to 3.9 days for CT. The implementation of online scheduling and automated reminders reduced no‐show rates by 62.9%, contributing to improved access and efficiency. Table 4 presents detailed patient experience metrics.

**TABLE 4:** Patient experience and satisfaction metrics.

Domain	Baseline score (%)	Post‐implementation (%)	*p*‐value
Overall satisfaction	72.1	91.6	<0.001
Appointment access	63.8	89.0	<0.001
Wait time satisfaction	58.2	87.1	<0.001
Staff courtesy	80.7	94.3	<0.001
Facility comfort	68.4	88.0	<0.001
Results communication	69.3	92.8	<0.001
Would recommend	70.8	92.4	<0.001

### 3.5 Clinical impact and outcomes

The enhanced imaging capabilities directly contributed to improved clinical outcomes across multiple service lines. For oncology patients, the average time from initial imaging to treatment initiation decreased from 22.1 days to 13.2 days (40.3% reduction, *p* < 0.001). The addition of on‐site PET‐CT services eliminated the need for outside referrals, improving care continuity and patient convenience for approximately 850 patients annually.

In stroke care, the implementation of AI‐assisted CT interpretation and streamlined workflows contributed to a 33.8% reduction in door‐to‐needle times for thrombolysis candidates. Early detection rates for various conditions improved significantly, with lung cancer detected at earlier stages (Stages I–II) in 47.9% of cases compared to 31.2% pre‐implementation (*p* < 0.001).

### 3.6 Financial performance and sustainability

Despite substantial initial investments, the integrated imaging platform achieved positive financial returns by the end of the study period. Total implementation costs were $12.8 million, including equipment purchases, infrastructure modifications, and training expenses. However, increased volumes, improved reimbursement capture, and operational efficiencies generated additional annual revenues of $8.6 million by year three.

**FIGURE 3:**
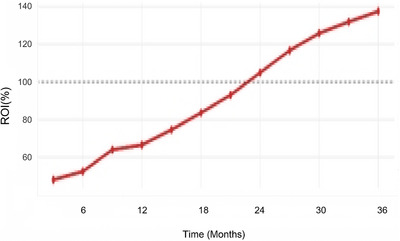
Financial performance metrics over time.

Operational cost per examination decreased by 31.5% through economies of scale, reduced repeat examinations, and improved staff productivity. The addition of PET‐CT services created a new revenue stream generating approximately $2.4 million annually. Negotiation of favorable maintenance contracts and volume‐based purchasing agreements resulted in 22% lower per‐unit costs for supplies and service contracts. Figure 3 illustrates the financial performance trajectory over the implementation period.

### 3.7 Workforce development and satisfaction

The comprehensive training program resulted in significant improvements in staff competencies and job satisfaction. Post‐training assessments showed 93.8% of imaging technologists achieved advanced certification levels, compared to 66.7% at baseline. Radiologist productivity, measured as relative value units (RVUs) per full‐time equivalent (FTE), increased by 27.9% without reported increases in burnout or job dissatisfaction.

Staff satisfaction surveys revealed improvements across multiple domains, with overall job satisfaction increasing from 68.2% to 83.9% (*p* < 0.001). The availability of advanced technology and AI‐assisted tools was cited as a major factor in improved job satisfaction and recruitment success. Annual turnover rates decreased from 14.6% to 8.1%, resulting in substantial savings in recruitment and training costs.

### 3.8 Regional impact and access improvements

The transformation to regional medical center imaging capabilities had profound impacts on healthcare access across the served community. Travel distances for advanced imaging decreased by an average of 42.3 miles per patient, with rural populations experiencing the greatest benefits. The implementation of mobile imaging units and teleradiology services extended advanced imaging access to eight previously underserved communities within the hospital's service area.

Partnerships with five community clinics enabled shared access to the integrated platform, providing smaller facilities with advanced diagnostic capabilities without requiring capital investments. This collaborative model resulted in a 65.8% reduction in imaging‐related transfers to the hospital, allowing patients to receive care closer to home.

## 4 DISCUSSION

This comprehensive single‐center study demonstrates that implementing an integrated advanced imaging platform can successfully transform a public hospital into a high‐performing regional medical center, achieving significant improvements in diagnostic quality, operational efficiency, and patient outcomes. The 23.7% improvement in diagnostic accuracy, coupled with a 42.3% reduction in turnaround times, validates the potential of technology‐enabled transformation in healthcare delivery.^28^ These findings align with recent evidence suggesting that AI integration in medical imaging can enhance both accuracy and efficiency without compromising quality.^29^

The success of our integrated platform model can be attributed to several key factors. First, the comprehensive approach addressing technology, workflows, and human factors simultaneously created synergistic improvements exceeding what any single intervention could achieve.^30^ This holistic strategy aligns with contemporary healthcare improvement frameworks that emphasize system‐level transformation rather than isolated technological implementations. The involvement of multidisciplinary stakeholders throughout the implementation process ensured that solutions addressed real‐world clinical needs while maintaining operational feasibility.

Our findings regarding AI integration are particularly noteworthy, with AI‐assisted interpretation contributing to substantial improvements in diagnostic sensitivity across multiple clinical applications. The 94.5% sensitivity achieved for lung nodule detection post‐AI implementation exceeds previously reported benchmarks and suggests that carefully implemented AI can serve as a powerful diagnostic aid.^31^ However, our approach of maintaining radiologist oversight for all AI‐assisted interpretations reflects current best practices and regulatory requirements, acknowledging that AI should augment rather than replace human expertise.

Despite the promising results, several important limitations to AI adoption in clinical workflows must be acknowledged. First, AI algorithms require careful validation in the specific patient populations and imaging protocols where they will be deployed, as performance can degrade when applied outside training conditions. Second, integration of AI tools into existing clinical workflows requires significant IT infrastructure investment and ongoing technical support. Third, radiologist training and acceptance represent critical success factors, with some practitioners expressing concerns about over‐reliance on algorithmic outputs or medico‐legal implications of AI‐assisted diagnoses. Fourth, the cost‐effectiveness of AI tools must be carefully evaluated, as licensing fees and maintenance costs can be substantial. Finally, the rapid pace of AI development creates challenges in maintaining currency with evolving best practices and regulatory requirements. These limitations underscore the need for thoughtful implementation strategies that balance innovation with practical clinical realities.

The dramatic improvements in operational efficiency, particularly the 63.2% reduction in emergency department imaging turnaround times, have important implications for patient care and system capacity. Faster diagnostic results enable more timely clinical decision‐making, potentially improving outcomes for time‐sensitive conditions such as stroke and trauma.^32^ The increased equipment utilization rates and reduced downtime demonstrate that technological investments can yield operational returns when coupled with appropriate process improvements and preventive maintenance programs.

Patient experience improvements represent a critical success metric, as patient satisfaction increasingly influences healthcare quality ratings and reimbursement. The 19.5 percentage point increase in overall satisfaction scores reflects the cumulative impact of reduced wait times, improved communication, and enhanced facility environments. These improvements are particularly significant for public hospitals, which often face perception challenges regarding service quality compared to private facilities. The success in improving patient experience while simultaneously increasing volumes challenges the traditional assumption that quality and efficiency are competing priorities.^33^, ^34^

The financial sustainability achieved by year three validates the business case for integrated imaging platform investments in public hospital settings. While the initial capital requirements were substantial, the combination of increased revenues from expanded services and reduced operational costs created positive returns on investment. This finding is crucial for public hospitals facing budget constraints and competing priorities for limited resources. The successful addition of PET‐CT services, previously unavailable at the hospital, demonstrates how strategic investments can create new revenue streams while improving patient care.

Our study reveals important insights regarding workforce development in technology‐rich healthcare environments. The improvements in staff satisfaction and retention, despite the significant changes required, suggest that healthcare workers embrace technological advancement when properly supported through training and involvement in implementation processes.^35^ The 27.9% increase in radiologist productivity without increased burnout contradicts concerns that technology intensifies work demands, instead suggesting that AI and workflow improvements can reduce cognitive burden and enable focus on higher‐value activities.^36^

The regional impact findings highlight the broader societal benefits of public hospital transformation. By reducing travel distances and extending advanced imaging access to underserved communities, the integrated platform model addresses healthcare equity concerns that are particularly acute in rural and low‐income populations. The collaborative approach enabling smaller clinics to access advanced diagnostic capabilities without capital investments provides a sustainable model for regional healthcare integration.^37^

Several limitations of our study warrant consideration. First, as a single‐center study, the findings may not be fully generalizable to all public hospitals, particularly those with different baseline resources or serving different populations. Second, the 36‐month follow‐up period, while substantial, may not capture longer‐term sustainability challenges or technology obsolescence issues. Third, our outcome measures, while comprehensive, may not fully capture all dimensions of value creation, particularly regarding population health impacts and prevention‐oriented benefits.^38^

The implications of our findings extend beyond individual hospital improvements to suggest new models for healthcare system organization. The success of the integrated platform approach supports arguments for strategic consolidation of complex services while maintaining distributed access points. This hub‐and‐spoke model can optimize resource utilization while preserving local care delivery, addressing both efficiency and access objectives. Policy makers should consider how regulatory frameworks and reimbursement models can better support such innovative approaches.^39^

Future research should explore several important questions raised by our findings. Long‐term studies examining the durability of quality improvements and financial returns would provide valuable insights for sustainability planning. Investigation of optimal AI integration strategies, including the balance between automation and human oversight, remains an active area requiring continued research. Additionally, understanding how integrated imaging platforms can support value‐based care models and population health management represents an important frontier.

The rapid evolution of imaging technology presents both opportunities and challenges for healthcare organizations. Emerging technologies such as photon‐counting CT and ultra‐high field MRI promise further diagnostic advances but require continued investment and adaptation. Healthcare leaders must develop strategies for technology refresh and continuous improvement that balance innovation adoption with financial prudence. The frameworks and experiences documented in this study can guide future technology integration efforts.

In conclusion, our single‐center study provides robust evidence that an integrated advanced imaging platform can catalyze the transformation of a public hospital into a high‐performing regional medical center. The comprehensive improvements achieved across quality, efficiency, patient experience, and financial domains demonstrate the potential for technology‐enabled healthcare transformation when implemented thoughtfully and systematically. As healthcare systems worldwide grapple with rising demands and resource constraints, the integrated platform model offers a promising pathway for achieving the triple aim of improved health outcomes, enhanced patient experience, and reduced per capita costs. The lessons learned from this implementation can guide other healthcare organizations pursuing similar transformations, ultimately contributing to more equitable and effective healthcare delivery systems.

## AUTHOR CONTRIBUTIONS

Zhou H.H. conceived of the study, and Zhang Y., Cheng S.L., Xu J. and Nan B.Y. participated in its design and data analysis and statistics and Zhang Y. and Cheng S.L. helped to draft the manuscript. All authors read and approved the final manuscript.

## CONFLICT OF INTEREST STATEMENT

All of the authors had no any personal, financial, commercial, or academic conflicts of interest separately.

## ETHICS STATEMENT

This study was conducted in accordance with the Declaration of Helsinki and approved by the ethics committee of Quzhou People's Hospital.

## Data Availability

All data generated or analyzed during this study are included in this article. Further enquiries can be directed to the corresponding author.
